# The Behavioral Recovery Outreach (BRO) Team: A Transitional Care Model for Veterans With Complex Care Needs

**DOI:** 10.1093/geroni/igab046.174

**Published:** 2021-12-17

**Authors:** Kathleen Matthews, Grant Bauste, Emily Luitjens

**Affiliations:** 1 VISN, 23, Des Moines, Iowa, United States; 2 Minneapolis VA Medical Center, Minneapolis, Minnesota, United States; 3 St. Cloud VA Health Care System, St. Cloud, Minnesota, United States

## Abstract

In 2012, VA Central Iowa developed a novel program known as the Behavioral Recovery Outreach (BRO) Team to address unmet needs of our aging Veteran population with complex medical, psychological, neurocognitive and behavioral concerns. BRO Teams provide evidence-informed treatments in inpatient VA settings, and transitional care/support post-discharge to ensure successful placement and stability in the community. We will discuss how implementation science informed the expansion of this model from a local pilot to a nationally disseminated program. We will explore the challenges of ensuring program fidelity while fostering innovation and adaptation. Given the challenges of national dissemination, we will highlight the predicted and unforeseen aspects of program evaluation and policy implications. Finally, we will discuss the impacts of the COVID-19 pandemic on delivery of care methods and community-based interactions, as well as how this program has improved the lives and quality of care for this high-risk Veteran population.

